# Draft Genome Sequence of the Haloacid-Degrading *Burkholderia caribensis* Strain MBA4

**DOI:** 10.1128/genomeA.00047-14

**Published:** 2014-02-20

**Authors:** Yanling Pan, Ka Fai Kong, Jimmy S. H. Tsang

**Affiliations:** Molecular Microbiology Laboratory, School of Biological Sciences, The University of Hong Kong, Hong Kong SAR, China

## Abstract

*Burkholderia caribensis* MBA4 was isolated from soil for its ability to utilize 2-haloacid. An inducible haloacid operon, encoding a dehalogenase and a permease, is mainly responsible for the biotransformation. Here, we report the draft genome sequence of this strain.

## GENOME ANNOUNCEMENT

Haloacetates such as monochloroacetate (MCA) are toxic and mutagenic and can be produced incidentally during disinfection of water. *Burkholderia caribensis* MBA4 is a Gram-negative bacterium that can utilize 2-haloacid as a growth substrate. This bacterium was characterized for its production of a dimeric hydrolytic dehalogenase (Deh4a) (1, 2) that removes the halogen from the carbon backbone. Here we describe the draft genome sequence of *Burkholderia caribensis* MBA4.

Analysis of *B. caribensis* MBA4 with pulsed-field gel electrophoresis showed that it has a genome size of more than 9 Mb with at least three replicons (data not shown). Whole-genomic sequencing was obtained with 454 GS FLX Titanium and Illumina HiSeq 2000. With low-quality short reads discarded, the 454 sequencing has 929,485 reads and 380,525,001 bp after trimming. Four sets of Illumina paired-end libraries with insert sizes of 100, 300, 500, and 2,000 bp were constructed and sequenced. After trimming and filtering, the four libraries have 37,483,321, 36,788,695, 23,594,431, and 12,689,821 high-quality paired-end reads, respectively. The average read lengths were 61, 61, 69, and 39 bp, respectively. The overall coverage is about 750-fold. Illumina paired-end and 454 reads were *de novo* assembled using CLC Genomic Workbench 6.0.1 (CLC bio, Aarhus, Denmark) with default settings. SSPACE basic 2.0 (3) was used to join contigs into scaffolds with information derived from paired-end reads. Moreover, 47,627 *de novo*-assembled transcripts from nine sets of RNA-seq data were mapped to the scaffolds to (i) remove some of the internal gaps, (ii) remove ambiguous base pairs, and (iii) join the scaffolds together. Standard PCR and Sanger-sequencing technology were applied to fill gaps inside the scaffolds. Multiplex PCR was used to amplify unknown regions between scaffolds, and some scaffolds were linked after subsequent cloning and sequencing. As a result, 14 scaffolds were obtained with 79 component contigs of >200 bp. Contig relationships were maintained in the GenBank submission by the inclusion of an AGP (A Golden Path) file. The total size of the contigs is 9,418,480 bp. The *N*_50_ of the contigs is 217,392 bp and the longest contig is 1,305,062 bp. The GC content was determined to be 62.48%, which is consistent with a result obtained from high-performance liquid chromatography (HPLC) analysis.

The draft genome was annotated automatically with the Rapid Annotations using Subsystems Technology (RAST) server (4) and the Prokaryotic Genomes Automatic Annotation Pipeline (PGAAP) from NCBI (5). The draft genome contains 9,082 genes, including 8 rRNA and 52 tRNA genes. Furthermore, there were 624 tandem repeats identified by Tandem Repeats Finder (6). Among the 9,022 protein-coding sequences, 76% were annotated as encoding known proteins while the remaining 24% encode hypothetical products. Among these RAST-annotated genes, 3,666 coding DNA sequences (CDS) were assigned to 27 subsystems. Analysis of the CDS with the KEGG Automatic Annotation Server (version 1.6a) (7) has specified 34 groups with 191 pathways.

### Nucleotide sequence accession numbers.

This whole-genome shotgun project has been deposited at DDBJ/EMBL/GenBank under the accession number AXDD00000000. The version described in this paper is version AXDD01000000.
